# Supplementary material for: WDR62 is required for centriole duplication in spermatogenesis and manchette removal in spermiogenesis
Source: Commun Biol. 2021 May 31;4:645. doi: 10.1038/s42003-021-02171-5 (PMC8167107; doi:10.1038/s42003-021-02171-5)
Supplement: Supplementary file 2 — Supplementary Information [file 42003_2021_2171_MOESM2_ESM.pdf]

Supplementary Figures and Table for

WDR62 is required for centriole duplication in spermatogenesis and manchette removal in spermiogenesis

Uda Y. Ho[1*], Chun-Wei Allen Feng[1], Yvonne Y. Yeap[1], Amanda L. Bain[2], Zhe Wei[3], Belal Shohayeb[1], Melissa E. Reichelt[1], Hayden Homer[3], Kum Kum Khanna[2], Josephine Bowles[1] and Dominic C.H. Ng[1*]

Affiliations
[1.] School of Biomedical Sciences, Faculty of Medicine, The University of Queensland, Brisbane, Australia
[2] QIMR Berghofer Medical Research Institute, Brisbane, Australia
[3.] UQ Centre for Clinical Research, Faculty of Medicine, The University of Queensland, Brisbane, Australia
* Corresponding authors: Uda Ho u.ho@uq.edu.au and Dominic Ng d.ng1@uq.edu.au

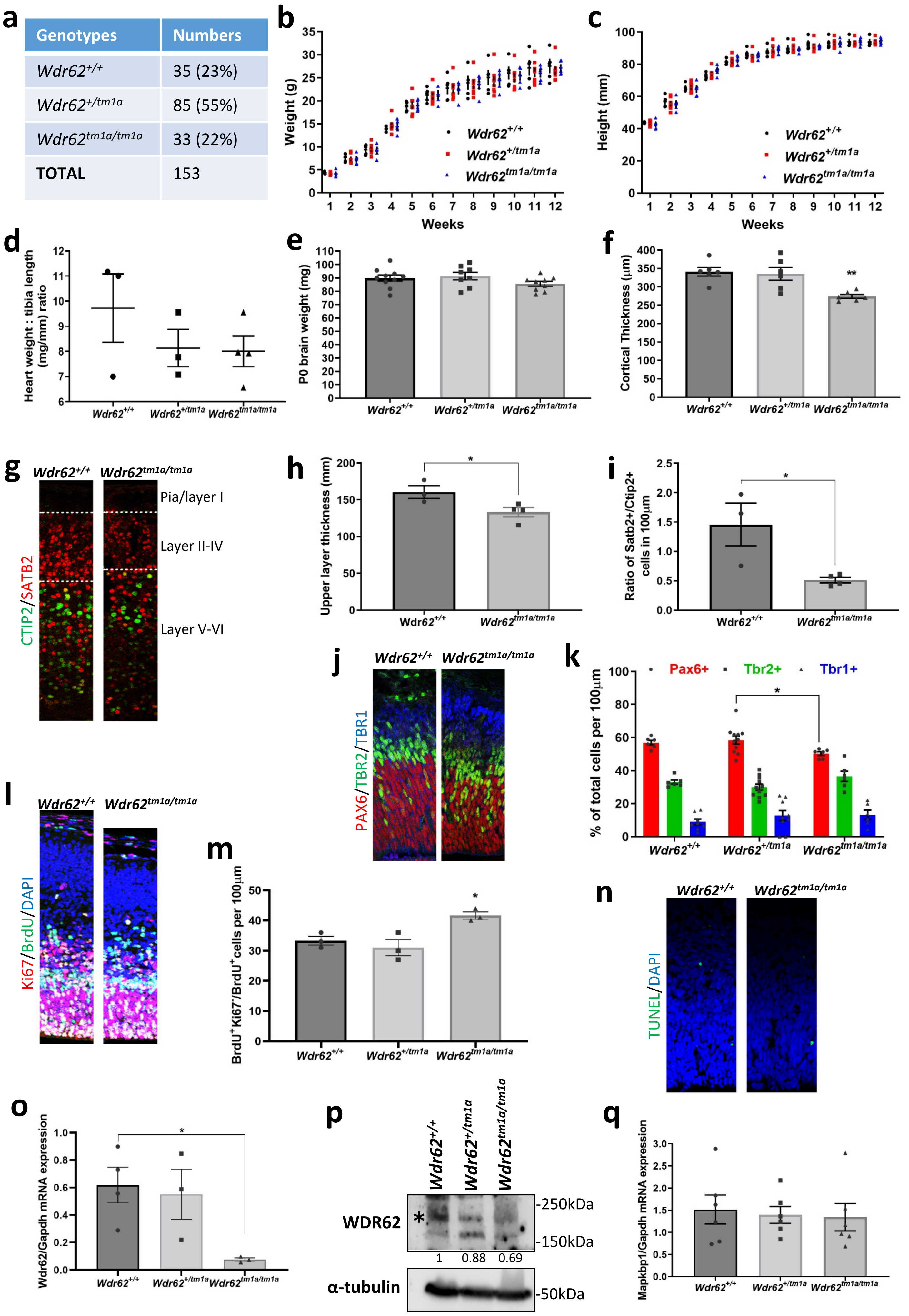

a
Genotypes | Numbers
Wdr62+/+ | 35 (23%)
Wdr62+/tm1a | 85 (55%)
Wdr62tm1a/tm1a | 33 (22%)
TOTAL | 153
b
Weight (g)
Weeks
Wdr62+/+
Wdr62+/tm1a
Wdr62tm1a/tm1a
c
Height (mm)
Weeks
Wdr62+/+
Wdr62+/tm1a
Wdr62tm1a/tm1a
d
Heart weight : tibia length (mg/mm) ratio
Wdr62+/+ Wdr62+/tm1a Wdr62tm1a/tm1a
e
P0 brain weight (mg)
Wdr62+/+ Wdr62+/tm1a Wdr62tm1a/tm1a
f
Cortical Thickness (μm)
**
Wdr62+/+ Wdr62+/tm1a Wdr62tm1a/tm1a
g
Wdr62+/+ Wdr62tm1a/tm1a
CTIP2/SATB2
Pia/layer I
Layer II-IV
Layer V-VI
h
Upper layer thickness (mm)
*
Wdr62+/+ Wdr62tm1a/tm1a
i
Ratio of Satb2+/Ctip2+ cells in 100μm
*
Wdr62+/+ Wdr62tm1a/tm1a
j
Wdr62+/+ Wdr62tm1a/tm1a
PAX6/TBR2/TBR1
k
Pax6+ Tbr2+ Tbr1+
% of total cells per 100μm
*
Wdr62+/+ Wdr62+/tm1a Wdr62tm1a/tm1a
l
Wdr62+/+ Wdr62tm1a/tm1a
Ki67/BrdU/DAPI
m
BrdU+Ki67-/BrdU+cells per 100μm
*
Wdr62+/+ Wdr62+/tm1a Wdr62tm1a/tm1a
n
Wdr62+/+ Wdr62tm1a/tm1a
TUNEL/DAPI
o
Wdr62/Gapdh mRNA expression
*
Wdr62+/+ Wdr62+/tm1a Wdr62tm1a/tm1a
p
Wdr62+/+ Wdr62+/tm1a Wdr62tm1a/tm1a
WDR62
*
-250kDa
-150kDa
1 0.88 0.69
α-tubulin
-50kDa
q
Mapkbp1/Gapdh mRNA expression
Wdr62+/+ Wdr62+/tm1a Wdr62tm1a/tm1a

Supplementary Figure 1. The *Wdr62$^{tm1a/tm1a}$* mouse show normal growth but thinner cerebral cortex and premature neuroprogenitor cell differentiation.

a. The number and percentages of wildtype (*Wdr62$^{+/+}$*), heterozygous (*Wdr62$^{+/tm1a}$*) and homozygous (*Wdr62$^{tm1a/tm1a}$*) mice produced from a total of 30 litters. **b.c.** No difference in weight (g) (b) and height (mm) (c) is observed between *Wdr62$^{+/+}$* (n=5), *Wdr62$^{+/tm1a}$* (n=8) and *Wdr62$^{tm1a/tm1a}$* (n=5) mice. **d.** Normalised heart weight (mg) to tibia length (mm) ratios in *Wdr62$^{+/+}$*, *Wdr62$^{+/tm1a}$* and *Wdr62$^{tm1a/tm1a}$* adult littermates. **e.** P0 Brain weight. n = 10 *Wdr62$^{+/+}$*, 8 *Wdr62$^{+/tm1a}$* and 9 *Wdr62$^{tm1a/tm1a}$*. **f.** Quantification of overall cortical thickness from hematoxylin and eosin stained sections. N= 6 independent brains per genotype. Two-tailed student t-test, **p=0.0069. **g.** CTIP2 (layer II-IV; green) and SATB1 (layer V-VI; red) co-immunofluorescence on P0 brain sections. **h.** Upper layer thickness (SATB2+ layer II-IV) was reduced in *Wdr62$^{tm1a/tm1a}$* compared to *Wdr62$^{+/+}$*. Two-tailed student t-test, *p=0.0466. **i.** The ratio of SATB2+ (layer II-IV) to CTIP2+ (layer V-VI) cells was reduced in *Wdr62$^{tm1a/tm1a}$* compared to *Wdr62$^{+/+}$*. Two-tailed student t-test, *p=0.0286. **j.** PAX6 (red)/TBR2 (green)/TBR1 (blue) co-immunofluorescence on *Wdr62$^{+/+}$* and *Wdr62$^{tm1a/tm1a}$* E14.5 brain sections. **k.** *Wdr62$^{tm1a/tm1a}$* E14.5 brains show a significant lower % of PAX6+ (apical progenitor) cells in 100µm^2. Two-way ANOVA, *p=0.0431. **l.** Cell cycle exit experiment, Ki67 (red) and BrdU (green) co-immunofluorescence on *Wdr62$^{+/+}$* and *Wdr62$^{tm1a/tm1a}$* E16.5 brain sections. **m.** Quantification of L in 100µm^2. *Wdr62$^{tm1a/tm1a}$* E16.5 brains show a significant higher % of BrdU$^+$Ki67$^-$ cells (cell cycle exited cells) in 100µm^2. n = 3 *Wdr62$^{+/+}$*, 3 *Wdr62$^{+/tm1a}$* and 3 *Wdr62$^{tm1a/tm1a}$*. All error bars represent S.E.M. Two-tailed student T-test, *p=0.0115. **n.** TUNEL staining (green) in *Wdr62$^{+/+}$* and *Wdr62$^{tm1a/tm1a}$* E14.5 brain coronal sections. Slides were counterstained with DAPI (blue). Scale bar represents 25µm. **o.** Wdr62 qRT-PCR shows reduced Wdr62 mRNA expression in *Wdr62$^{tm1a/tm1a}$* P0 brains compared to control littermates. Two-tailed student t-test, *p=0.0170. n=6 brains per genotype. **p.** WDR62 western analysis shows reduced WDR62 protein level in *Wdr62$^{tm1a/tm1a}$* P0 brains compared to control littermates. Normalised band quantification as shown. **q.** Mapkbp1 qRT-PCR shows unaltered Mapkbp1 mRNA expression in *Wdr62$^{tm1a/tm1a}$* P0 brains compared to control littermates. n=6 brains per genotype. All error bars represent S.E.M.

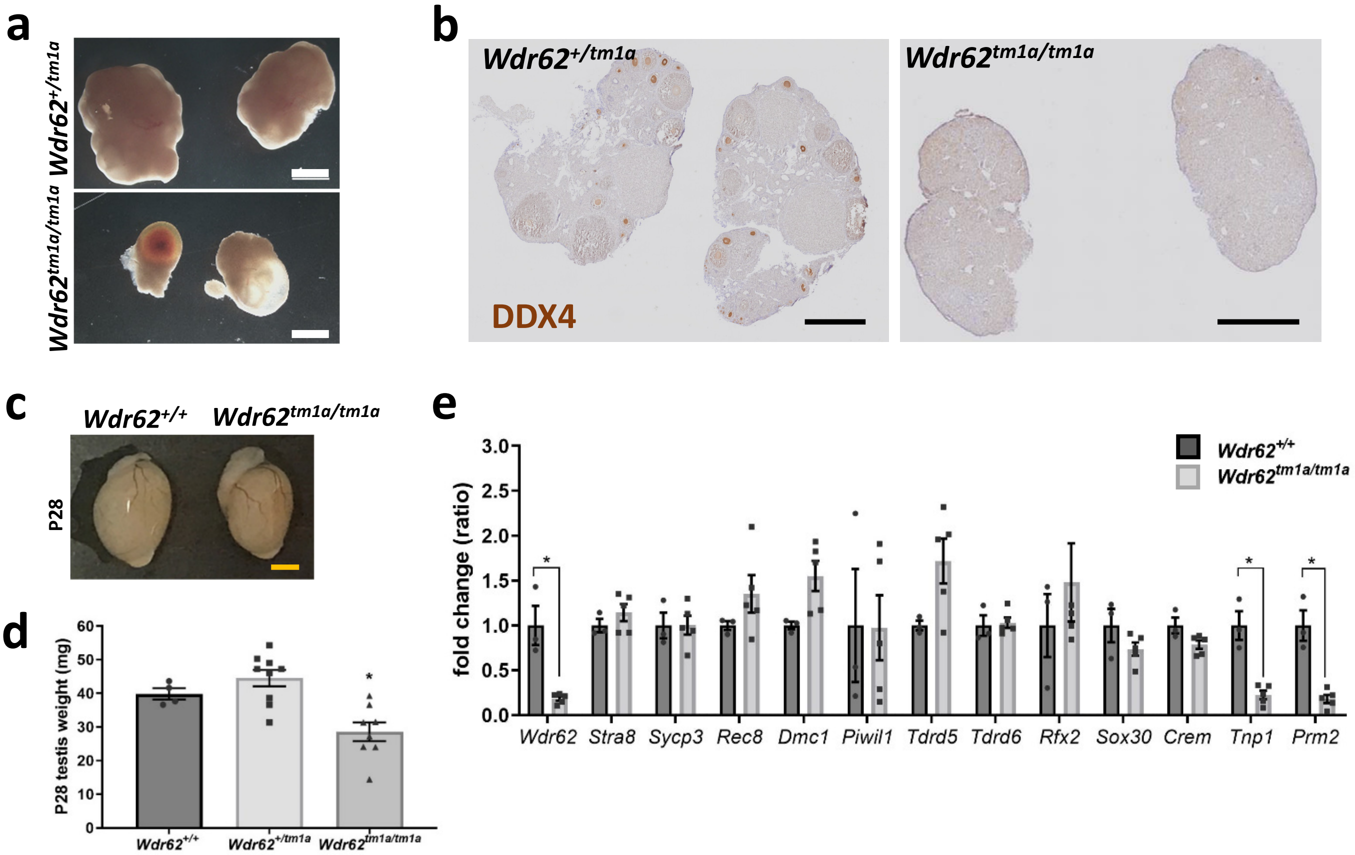

Supplementary Figure 2. WDR62 deficiency leads to smaller ovaries and testis.
a. Ovaries from 8 week old female *Wdr62*$^{+/tm1a}$ and *Wdr62*$^{tm1a/tm1a}$ littermates. Scale bar represents 1mm. **b.** Representative images of DDX4 immunohistochemistry, which shows DDX4+ germ cells presented in *Wdr62*$^{+/tm1a}$ but not in *Wdr62*$^{tm1a/tm1a}$ 8 week old ovaries. Scale bar represents 500µm. **c.** Testes from *Wdr62*$^{+/+}$ and *Wdr62*$^{tm1a/tm1a}$ littermates collected at post-natal day 28 (P28). Scale bar represents 1mm.**d.** Testis weight for *Wdr62*$^{+/+}$, *Wdr62*$^{+/tm1a}$ and *Wdr62*$^{tm1a/tm1a}$ P28 testis. n=4 *Wdr62*$^{+/+}$, 9 *Wdr62*$^{+/tm1a}$ and 8 *Wdr62*$^{tm1a/tm1a}$ P28 testes. Two-tailed unpaired student t-test, *p=0.0230. **e.** qRT-PCR of meiotic initiation (Stra8), spermatogenesis (Sycp3, Rec8, Dmc1, Piwi1, Tdrd5, Tdrd6) and round spermatid (Rfx2, Sox30, Crem, Tnp1, Prm2) markers in *Wdr62*$^{+/+}$ and *Wdr62*$^{tm1a/tm1a}$ P28 testes. N=3 *Wdr62*$^{+/+}$ and 5 *Wdr62*$^{tm1a/tm1a}$ P28 testes. Mean +/- S.E.M. Two-tailed unpaired student t-test, *p=0.0025 (Wdr62), p=0.0011 (Tnp1), p=0.0010 (Prm2).

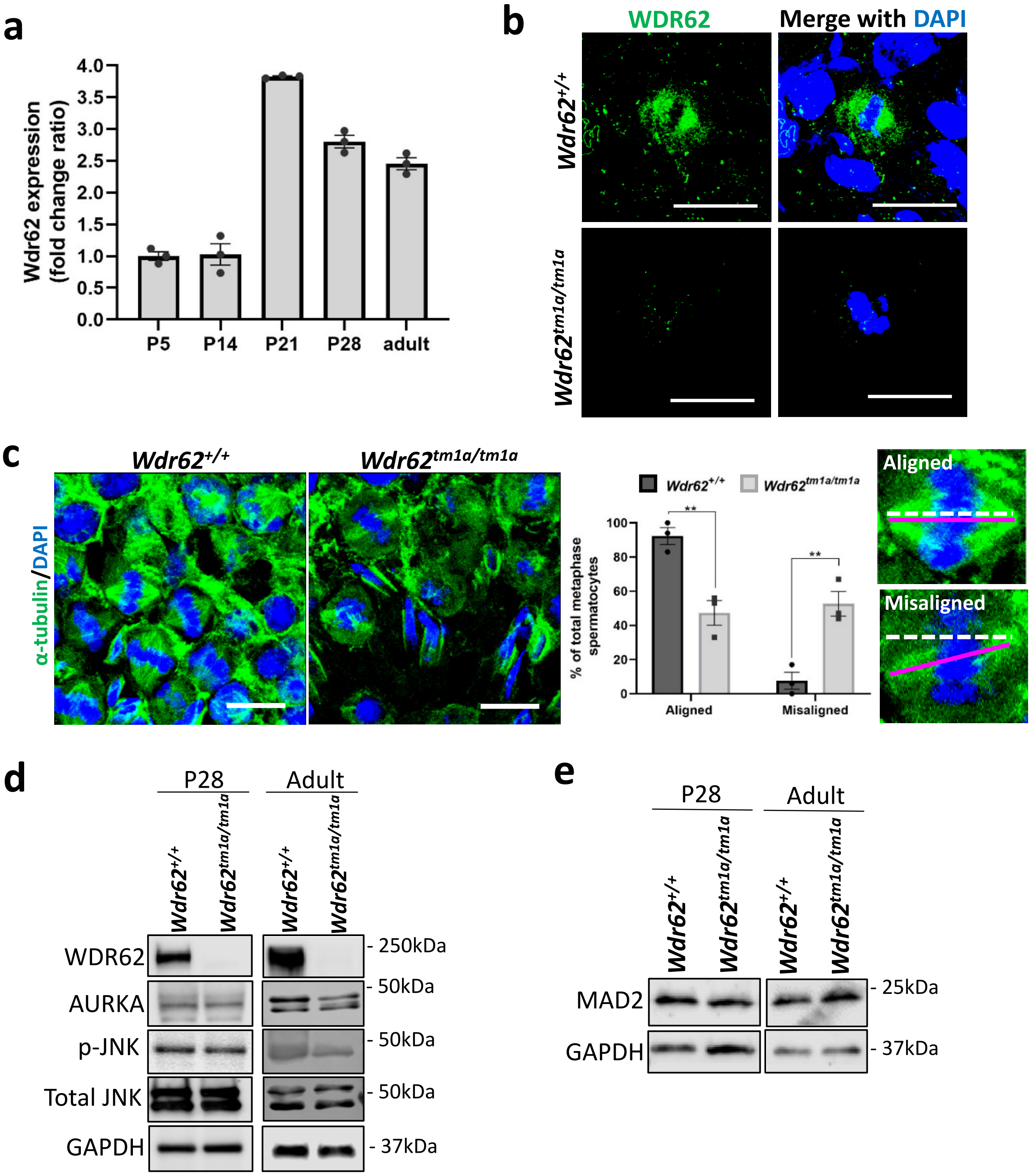

Supplementary Figure 3. WDR62 deficient spermatocytes show misaligned meiotic spindles.

a. qRT-PCR shows Wdr62 mRNA expression (fold change relative to P5) in P5, P14, P21, P28 and adult *Wdr62*$^{+/+}$ testis. n=3 *Wdr62*$^{+/+}$ testes per timepoint. Error bars represent S.E.M. **b.** WDR62 (green) immunofluorescence on *Wdr62*$^{+/+}$ and *Wdr62*$^{tm1a/tm1a}$ adult spermatocyte spreads. Metaphase spermatocytes are shown here. Sections are counterstained with DAPI (blue). Scale bar represents 20µm. **c.** α-tubulin (green) immunofluorescence on *Wdr62*$^{+/+}$ and *Wdr62*$^{tm1a/tm1a}$ adult testis sections. Sections are counterstained with DAPI (blue). Scale bar represents 10µm. The percentage of aligned and misaligned bipolar meiotic spindles were quantified in metaphase spermatocytes. Examples of metaphase spermatocytes with aligned or misaligned bipolar meiotic spindles are illustrated. White dotted line = expected bipolar spindle alignment; pink line = actual spindle alignment. n=57 *Wdr62*$^{+/+}$ and 43 *Wdr62*$^{tm1a/tm1a}$ spermatocytes from 3 independent testes per genotype. Two-way ANOVA, **p=0.0018. **c.** WDR62, AURKA, p-JNK and total JNK western analyses of *Wdr62*$^{+/+}$ and *Wdr62*$^{tm1a/tm1a}$ testis lysates. **d.** MAD2 western analysis in *Wdr62*$^{+/+}$ and *Wdr62*$^{tm1a/tm1a}$ P28 and adult testis. GAPDH was used as loading control in c and d. See Supplementary Figure 6 for western blots and band quantification.

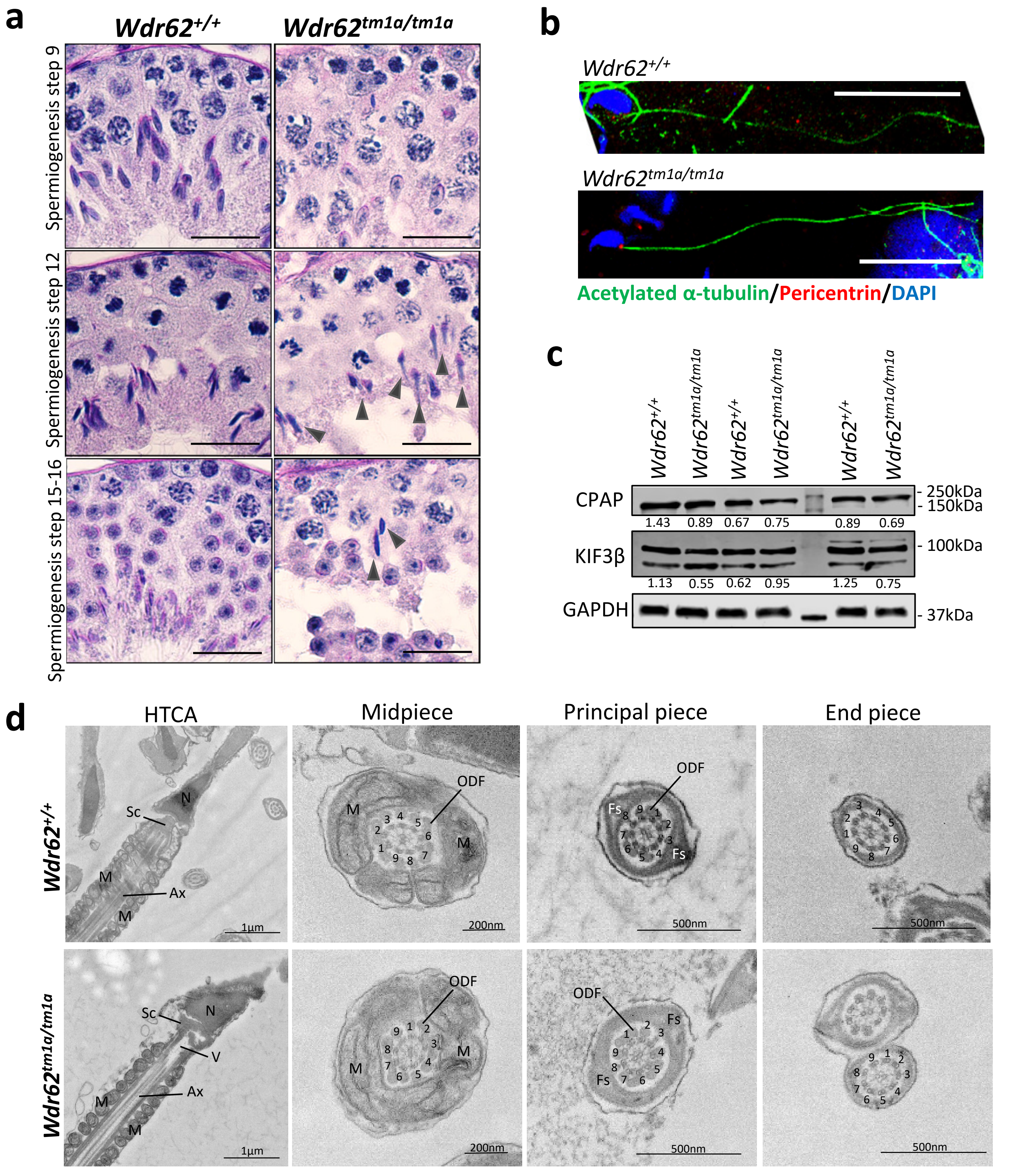

Supplementary Figure 4. WDR62 deficiency caused misshapen sperm head but did not affect acrosome, axoneme and flagellum formation.

a. PAS-H staining of adult *Wdr62*$^{+/+}$ and *Wdr62*$^{tm1a/tm1a}$ testis showing spermiogenesis step 9, 12 and 15-16 spermatids with normal acrosome formation. Red = acrosome cap, purple = nucleus/chromosomes, gray arrowhead = misshapen spermatid head. Scale bar represents 20µm. **b.** Acetylated α-tubulin (green) and Pericentrin (red) co-immunofluorescence on spermatocyte spreads show intact flagellum in both *Wdr62*$^{+/+}$ and *Wdr62*$^{tm1a/tm1a}$ elongated spermatids. Scale bar represents 20µm. **c.** Western analysis show similar CPAP and KIF3β protein expression in both *Wdr62*$^{+/+}$ and *Wdr62*$^{tm1a/tm1a}$ testis lysates, indicating normal cilium/flagellum elongation. GAPDH is used as loading control. **d.** Transmission electron microscopy of caudal epididymal spermatozoa showing normal HTCA, mitochrondrial sheath and fibrous sheath surrounding the axoneme in the midpiece and principal piece of the sperm flagellum respectively in both *Wdr62*$^{+/+}$ and *Wdr62*$^{tm1a/tm1a}$. Normal 9+2 microtubule arrangement of the axoneme are seen in the midpiece, principal piece and end piece of *Wdr62*$^{+/+}$ and *Wdr62*$^{tm1a/tm1a}$ sperm flagellum. HTCA = head-tail coupling apparatus; M = mitochrondria; N = nucleus/sperm head; Sc = segmented columns; V = centriolar vault; Ax = axoneme; Fs = fibrous sheath; ODF = outer dense fibre; the outer nine fused pairs of microtubule of the axoneme are labelled 1 to 9. Scale bars as indicated.

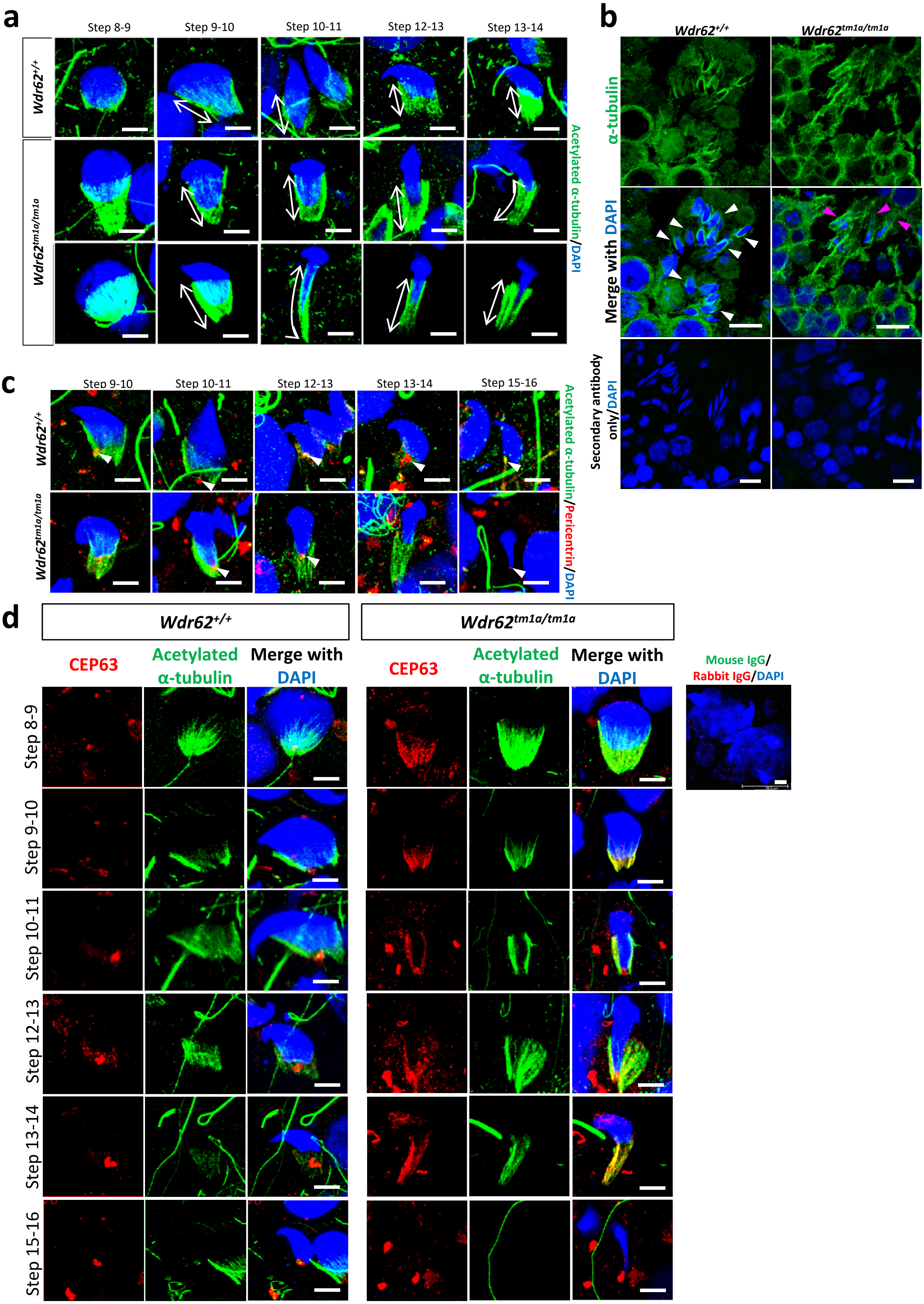
a
Step 8-9
Step 9-10
Step 10-11
Step 12-13
Step 13-14
Wdr62+/+
Wdr62tm1a/tm1a
Acetylated α-tubulin/DAPI
b
Wdr62+/+
Wdr62tm1a/tm1a
α-tubulin
Merge with DAPI
Secondary antibody only/DAPI
c
Step 9-10
Step 10-11
Step 12-13
Step 13-14
Step 15-16
Wdr62+/+
Wdr62tm1a/tm1a
Acetylated α-tubulin/Pericentrin/DAPI
d
Wdr62+/+
Wdr62tm1a/tm1a
CEP63
Acetylated α-tubulin
Merge with DAPI
Mouse IgG/
Rabbit IgG/DAPI
Step 8-9
Step 9-10
Step 10-11
Step 12-13
Step 13-14
Step 15-16

Supplementary Figure 5. WDR62 deficiency display manchette removal defect leading to misshapen sperm head.

a. Acetylated α-tubulin (green) immunofluorescence on adult spermatocyte spreads show manchette formation and deformation during step 8-14 spermiogenesis. White arrow indicates the length of manchette in relation to Figure 5e. **b**. α-tubulin (green) immunofluorescence on adult testis sections show elongated manchette in step 12 spermatids of *Wdr62*$^{tm1a/tm1a}$ compared to *Wdr62*$^{+/+}$. White arrowheads indicate normal manchette, pink arrowheads indicate elongated manchette. Secondary antibody only is used as negative control. Scale bars represent 10µm. **c.** Acetylated α-tubulin (green) and Pericentrin (red) co-immunofluorescence on adult spermatocyte spreads showing step 9 to 16 spermatids. White arrowheads indicate Pericentrin accumulation at the pericentriolar matrix. **d.** Acetylated α-tubulin (green) and CEP63 (red) co-immunofluorescence show CEP63 accumulation at the caudal side of sperm head in step 8-16 *Wdr62*$^{+/+}$spermatids. However, CEP63 co-localises with the manchettein step 10-14 *Wdr62*$^{tm1a/tm1a}$ spermatids in addition to accumulation at the caudal side of the spermatid head, indicating reduced or delayed CEP63 accumulation caused by WDR62 deficiency. Mouse and rabbit IgG antibodies are used as negative control. All slides were counterstained with DAPI (blue). Scale bars represent 5µm in a, c and d.

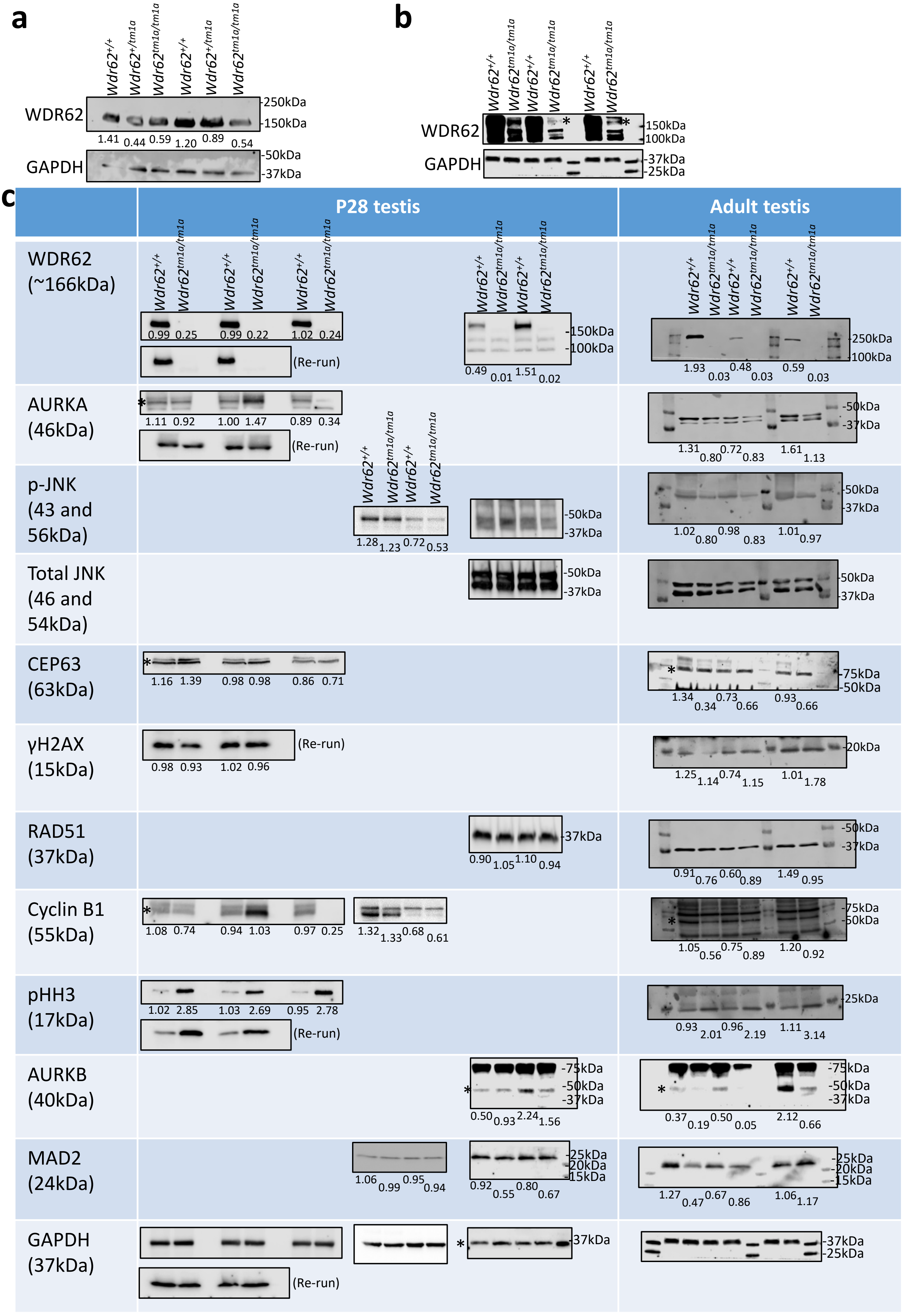
a
$Wdr62^{+/+}$
$Wdr62^{+/tm1a}$
$Wdr62^{tm1a/tm1a}$
WDR62
250kDa
150kDa
1.41 0.44 0.59 1.20 0.89 0.54
GAPDH
50kDa
37kDa
b
WDR62
150kDa
100kDa
GAPDH
37kDa
25kDa
c
P28 testis
Adult testis
WDR62 (~166kDa)
0.99 0.25 0.99 0.22 1.02 0.24
(Re-run)
0.49 0.01 1.51 0.02
1.93 0.03 0.48 0.03 0.59 0.03
AURKA (46kDa)
1.11 0.92 1.00 1.47 0.89 0.34
1.31 0.80 0.72 0.83 1.61 1.13
p-JNK (43 and 56kDa)
1.28 1.23 0.72 0.53
1.02 0.80 0.98 0.83 1.01 0.97
Total JNK (46 and 54kDa)
CEP63 (63kDa)
1.16 1.39 0.98 0.98 0.86 0.71
1.34 0.34 0.73 0.66 0.93 0.66
75kDa
γH2AX (15kDa)
0.98 0.93 1.02 0.96
1.25 1.14 0.74 1.15 1.01 1.78
20kDa
RAD51 (37kDa)
0.90 1.05 1.10 0.94
0.91 0.76 0.60 0.89 1.49 0.95
Cyclin B1 (55kDa)
1.08 0.74 0.94 1.03 0.97 0.25
1.32 1.33 0.68 0.61
1.05 0.56 0.75 0.89 1.20 0.92
pHH3 (17kDa)
1.02 2.85 1.03 2.69 0.95 2.78
0.93 2.01 0.96 2.19 1.11 3.14
AURKB (40kDa)
0.50 0.93 2.24 1.56
0.37 0.19 0.50 0.05 2.12 0.66
MAD2 (24kDa)
1.06 0.99 0.95 0.94
0.92 0.55 0.80 0.67
1.27 0.47 0.67 0.86 1.06 1.17
15kDa
GAPDH (37kDa)

Supplementary Figure 6. Western blots and band quantification in this study.
The normalised value of each band (band value/GAPDH value of the same sample from the same blot) is shown directly underneath each band. **a.** WDR62 western analysis in P0 brain samples (related to Supplementary Figure 1p). **b.** WDR62 western analysis in adult testes with increased exposure to show the WDR62 band in $Wdr62^{tm1a/tm1a}$. **c.** Western blots using P28 and adult testes lysates performed in this study (related to Figure 2d, 4b and Supplementary Figure 3c, 3d, 4c).
*indicates correct band.

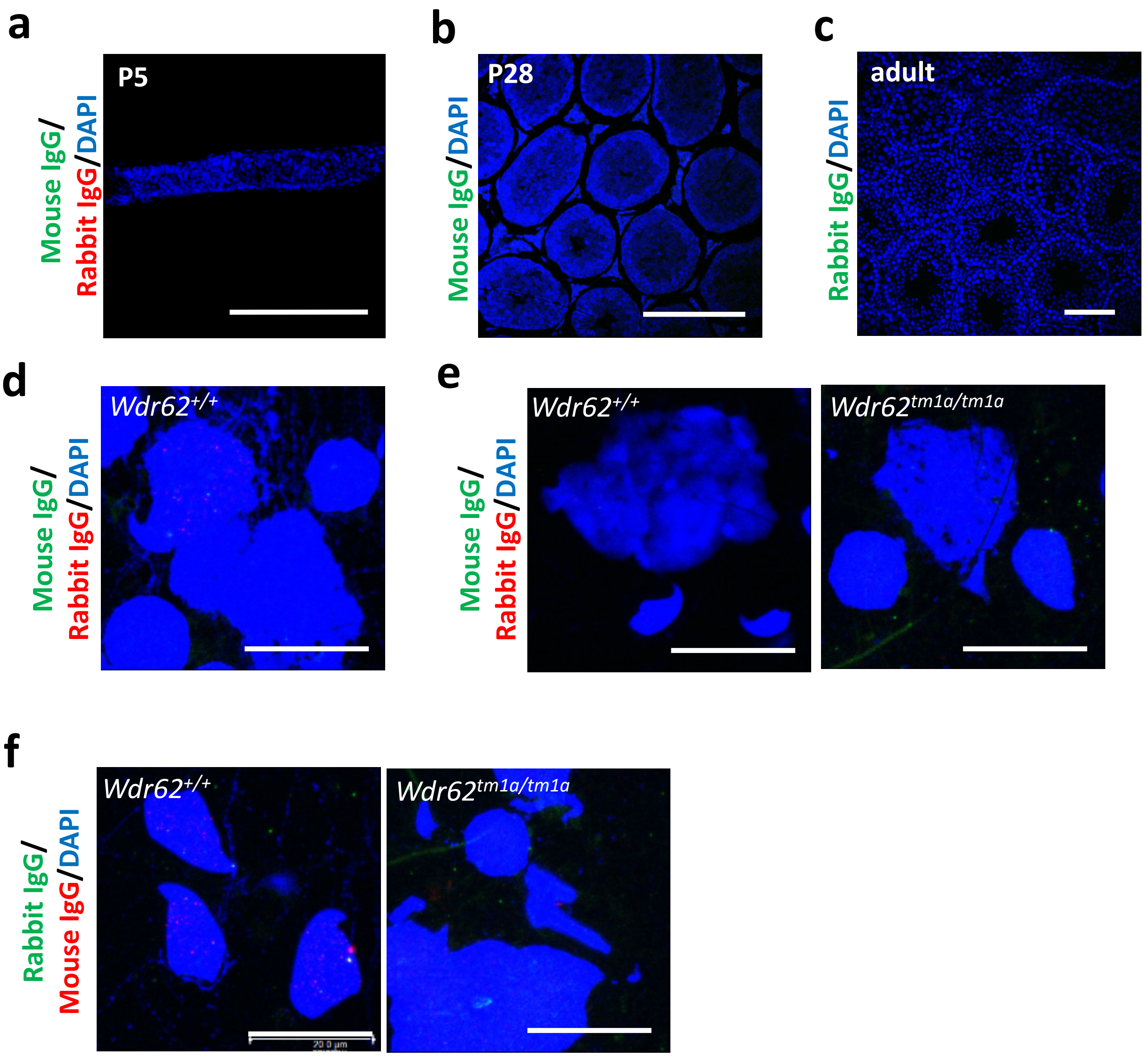

Supplementary Figure 7. Negative controls of the immunofluorescence experiments in this study. **a.** Mouse IgG (green) and Rabbit IgG (red) control of DDX4 and STRA8 whole mount co-immunofluorescence of P5 seminiferous tubules in Figure 2a. Scale bar represents 200µm. **b.** Mouse IgG (green) control of DDX4 staining on P28 testis sections in Figure 2a. Both DDX4 and IgG control staining on P21 and P28 sections were performed at the same time. Scale bar represents 200µm. **c.** Rabbit IgG (green) control of pHH3 staining on adult testis sections in Figure 3a. pHH3 staining on P28 and adult testis sections were performed at the same time. Scale bar represents 100µm. **d.** Mouse IgG (green) and Rabbit IgG (red) control of Centrin-SYCP3 and Pericentrin co-staining in Figure 4a. Scale bar represents 20µm. **e.** Mouse IgG (green) and Rabbit IgG (red) control of Centrin-SYCP3 and CEP63 co-staining in Figure 4c. Scale bar represents 20µm. **f.** Rabbit IgG (green) and Mouse IgG (red) control staining of Katanin p60 or p80 and acetylated α-tubulin co-staining in Figure 6a and b. Scale bar represents 20µm. All seminiferous tubules, testis sections or spermatocyte spreads are counterstained with DAPI (blue).

Supplementary Table 1. Antibodies used for immunoblotting and immunofluorescence in this study.

Epitope	Host species	Company	Dilution	Application
WDR62 02B	Rabbit	Xu et al., 2014	1:50 (IF) 1:1000 (western)	Western, IF, spermatocyte spreads
PAX6	Mouse	DSHB PAX6S	1:200	IF
TBR2	Rat-AF488 conjugated	Life Technology 53-4875-82	1:200	IF
TBR1	Rabbit	Abcam ab31940	1:200	IF
CTIP2	Rat	Abcam ab18465	1:200	IF
SATB2	Rabbit	Abcam ab51502	1:400	IF
BrdU	Mouse	DSHB BRDUS	1:200	IF
Ki67	Rabbit	Cell Signaling 9129s	1:200	IF
pHH3	Rabbit	Millipore 06-570	1:400 (IF) 1:1000 (Western)	Western, IF
γH2AX	Rabbit	Cell Signaling 9718s	1:200 (IF) 1:1000 (Western)	Western, IF on spermatocyte spreads
SYCP3	Mouse	Abcam ab97672	1:200	IF, Spermatocyte spreads
STRA8 KLH	Rabbit	Abcam ab49405	1:200	IF
STRA8 (N-term)	Rabbit	Abcam ab49602	1:200	IF
DEAD-box helicase 4 (DDX4/MVH)	Mouse	Abcam ab27591	1:500	Whole mount IF, spermatocyte spreads
Aurora kinase A	Mouse	Abcam ab13824	1:200 (IF) 1:1000 (Western)	Spermatocyte spreads, Western
Phosphor JNK	Mouse	BD Pharmingen 612540	1:1000	Western
Total JNK	Mouse	BD Pharmingen 554285	1:1000	Western
Cyclin B1	Mouse	Cell Signaling 4135S	1:1000	Western
RAD51	Rabbit	Santa Cruz sc-8349	1:1000	Western
CEP63	Rabbit	Millipore 06-1292	1:200 (IF) 1:1000 (Western)	Western, IF, spermatocyte spreads
KIF3β	Rabbit	Santa Cruz sc-50456	1:1000	Western
CPAP/CENPJ	Rabbit	Proteintech 11517-1-AP	1:1000	Western
Centrin	Mouse	Millipore 04-1624	1:200	spermatocyte spreads
Pericentrin	Rabbit	Abcam ab4448	1:200	IF, spermatocyte spreads
α-tubulin	Mouse	Sigma T5168	1:200	IF
Acetylated α-tubulin	Mouse	Sigma T6793	1:200	IF, spermatocyte spreads
GAPDH	Rabbit	Santa Cruz sc-25778	1:1000	Western
Aurora kinase B/ARK2	Mouse	Santa Cruz sc-393357	1:1000	Western
MAD2	Mouse	Santa Cruz sc-47747	1:1000	Western
Katanin p60 (KATNA1)	Rabbit	Proteintech 17560-1-AP	1:200 (IF) 1:1000 (Western)	Spermatocyte spreads, Western
Katanin p80 (KATNB1)	Rabbit	Proteintech 14969-1-AP	1:200 (IF) 1:1000 (Western)	Spermatocyte spreads, Western
Mouse Alexa 488	Goat	Invitrogen A11001	1:300	Secondary antibody for IF
Mouse Alexa 555	Goat	Invitrogen A21424	1:300	Secondary antibody for IF
Rabbit Alexa 488	Goat	Invitrogen A11034	1:300	Secondary antibody for IF
Rabbit Alexa 555	Goat	Invitrogen A21428	1:300	Secondary antibody for IF
Rabbit Alexa 647	Goat	Invitrogen A27040	1:300	Secondary antibody for IF
Mouse HRP	Goat	Pierce PIE31430	1:5000	Secondary antibody for Western
Rabbit HRP	Goat	Pierce PIE31460	1:5000	Secondary antibody for Western
DAPI 5mg/mL	n/a	Sigma D8417	1:10,000	IF
IgG control	Rabbit	Santa Cruz sc-2027	1:200	IF, spermatocyte spreads
IgG control	Mouse	Santa Cruz sc-2025	1:200	IF, spermatocyte spreads
