# Supplementary material for: WDR62 is required for centriole duplication in spermatogenesis and manchette removal in spermiogenesis
Source: Commun Biol. 2021 May 31;4:645. doi: 10.1038/s42003-021-02171-5 (PMC8167107; doi:10.1038/s42003-021-02171-5)
Supplement: Supplementary file 3 — Description of Additional Supplementary Files [file 42003_2021_2171_MOESM3_ESM.pdf]

Description of Additional Supplementary Files

File name: Supplementary Data 1

Description: Source data for figures in a single excel file.
