# Supplementary material for: WDR62 is required for centriole duplication in spermatogenesis and manchette removal in spermiogenesis
Source: Commun Biol. 2021 May 31;4:645. doi: 10.1038/s42003-021-02171-5 (PMC8167107; doi:10.1038/s42003-021-02171-5)
Supplement: Supplementary file 5 — Reporting Summary [file 42003_2021_2171_MOESM5_ESM.pdf]

nature research

Corresponding author(s): Uda Ho and Dominic Ng

Last updated by author(s): Apr 22, 2021

Reporting Summary

Nature Research wishes to improve the reproducibility of the work that we publish. This form provides structure for consistency and transparency in reporting. For further information on Nature Research policies, see our Editorial Policies and the Editorial Policy Checklist.

Statistics

For all statistical analyses, confirm that the following items are present in the figure legend, table legend, main text, or Methods section.

n/a	Confirmed	
[ ]	[x]	The exact sample size (*n*) for each experimental group/condition, given as a discrete number and unit of measurement
[ ]	[x]	A statement on whether measurements were taken from distinct samples or whether the same sample was measured repeatedly
[ ]	[x]	The statistical test(s) used AND whether they are one- or two-sided<br>*Only common tests should be described solely by name; describe more complex techniques in the Methods section.*
[x]	[ ]	A description of all covariates tested
[x]	[ ]	A description of any assumptions or corrections, such as tests of normality and adjustment for multiple comparisons
[ ]	[x]	A full description of the statistical parameters including central tendency (e.g. means) or other basic estimates (e.g. regression coefficient) AND variation (e.g. standard deviation) or associated estimates of uncertainty (e.g. confidence intervals)
[x]	[ ]	For null hypothesis testing, the test statistic (e.g. *F*, *t*, *r*) with confidence intervals, effect sizes, degrees of freedom and *P* value noted<br>*Give P values as exact values whenever suitable.*
[x]	[ ]	For Bayesian analysis, information on the choice of priors and Markov chain Monte Carlo settings
[x]	[ ]	For hierarchical and complex designs, identification of the appropriate level for tests and full reporting of outcomes
[x]	[ ]	Estimates of effect sizes (e.g. Cohen's *d*, Pearson's *r*), indicating how they were calculated

Our web collection on statistics for biologists contains articles on many of the points above.

Software and code

Policy information about availability of computer code

Data collection	Quantitative real time PCR: QuantStudio 7 Flex system (ABI Systems); Immunofluorescence: LAS-X (Leica); Western: Odyssey-Fc (LI-COR).
Data analysis	QuantStudio software version 1.3; Microsoft Excel 2016; Graphpad Prism version 8.3; Image Studio Lite version 5.2; Leica Application Suite Advanced Fluorescence Lite (LAS AF Lite) software version 2.6.3 build 8173.

For manuscripts utilizing custom algorithms or software that are central to the research but not yet described in published literature, software must be made available to editors and reviewers. We strongly encourage code deposition in a community repository (e.g. GitHub). See the Nature Research guidelines for submitting code & software for further information.

Data

Policy information about availability of data

All manuscripts must include a data availability statement. This statement should provide the following information, where applicable:

- Accession codes, unique identifiers, or web links for publicly available datasets
- A list of figures that have associated raw data
- A description of any restrictions on data availability

All data generated and analysed in this study are included in this article and its Supplementary Information files.

Field-specific reporting

Life sciences study design

All studies must disclose on these points even when the disclosure is negative.

Sample size	No sample size calculation was taken to predetermine sample size. At least 3 animals per genotype were used per experiment to ensure reproducibility. The n number for each experiment is detailed in the figure legends.
Data exclusions	No data was excluded for analysis.
Replication	Experiments were replicated using at least 3 homozygous animals and their littermates. All attempts at replication were successful.
Randomization	Male mice of the correct genotypes were allocated for testis analysis, while female mice were allocated for ovary analysis. Mice were allocated for each experiments depending on availability and age. Each homozygous sample is compared to its control littermate.
Blinding	All experiments and analysis were performed in a blinded manner, with genotypes revealed at the graphing and/or figure compilation step.

Reporting for specific materials, systems and methods

We require information from authors about some types of materials, experimental systems and methods used in many studies. Here, indicate whether each material, system or method listed is relevant to your study. If you are not sure if a list item applies to your research, read the appropriate section before selecting a response.

Materials & experimental systems

n/a	Involved in the study	
☐	☒	Antibodies
☒	☐	Eukaryotic cell lines
☒	☐	Palaeontology and archaeology
☐	☒	Animals and other organisms
☒	☐	Human research participants
☒	☐	Clinical data
☒	☐	Dual use research of concern

Methods

n/a	Involved in the study	
☒	☐	ChIP-seq
☒	☐	Flow cytometry
☒	☐	MRI-based neuroimaging

Antibodies

Antibodies used	Please see Supplementary Table 1.
Validation	The antibodies used in this study have been previously used in Shohayeb et al., 2019 and Lim et al., 2016. Please see Supplementary Table 1 for applications/experiments and dilutions used.

Animals and other organisms

Policy information about studies involving animals; ARRIVE guidelines recommended for reporting animal research

Laboratory animals	Mus musculus (mouse), C57Bl/6, both males and females for timed matings, males for testis analysis, females for ovaries analysis.
Wild animals	The study did not involve wild animals.
Field-collected samples	The study did not involve samples collected from the field.
Ethics oversight	All animal experiments were approved by the Animal Ethics Committee at the University of Queensland.

Note that full information on the approval of the study protocol must also be provided in the manuscript.
